# Associations of triglyceride-glucose-related indices and cardiovascular health with retinal arteriosclerosis: a cross-sectional study

**DOI:** 10.3389/fpubh.2026.1766122

**Published:** 2026-03-16

**Authors:** Qingqing Zhang, Guoyu Wang, Hong Xu, Zixiang Li, Peng Gao, Jing Zheng, Suyun Jiang, Si Sun, Yucheng Wu, Jingyuan Cao, Ming Chu

**Affiliations:** 1 Department of Pan-Vascular Management Center, The Affiliated Taizhou People’s Hospital of Nanjing Medical University, Taizhou School of Clinical Medicine, Nanjing Medical University, Taizhou, Jiangsu, China; 2 Department of Endocrinology, The Affiliated Taizhou People’s Hospital of Nanjing Medical University, Taizhou School of Clinical Medicine, Nanjing Medical University, Taizhou, Jiangsu, China; 3 Department of Ophthalmology, The Affiliated Hospital of Yangzhou University, Yangzhou, Jiangsu, China; 4 Department of Cardiology, The Affiliated Taizhou People’s Hospital of Nanjing Medical University, Taizhou School of Clinical Medicine, Nanjing Medical University, Taizhou, Jiangsu, China

**Keywords:** cardiovascular health, insulin resistance, LE8, life’s essential 8, retinal arteriosclerosis, triglyceride-glucose index, TyG-related indices

## Abstract

**Objective:**

Retinal arteriosclerosis (RA) is a visible marker of early microvascular injury largely driven by cardiovascular risk factors. Insulin resistance, represented by the triglyceride-glucose (TyG) related indices, may serve as a key mediator linking cardiovascular health (CVH) to RA.

**Methods:**

In this cross-sectional study of 755 healthcare staff aged ≥35 years, RA was defined as Keith-Wagener-Barker grade ≥1 on fundus photography, representing early subclinical retinal arteriolar changes. CVH was assessed using Life’s Essential 8 (LE8). TyG-related indices, including TyG, TyG combined with body mass index (TyG-BMI), TyG combined with body roundness index (TyG-BRI), TyG combined with waist circumference (TyG-WC), and TyG combined with the waist-to-height ratio (TyG-WHtR), were calculated. Logistic regression and mediation analyses were performed to evaluate the associations of TyG-related indices with RA, and their mediating role in the LE8–RA relationship.

**Results:**

RA was present in 159 participants (21.1%). Higher TyG-related indices were significantly associated with an increased odds of RA. In fully adjusted models, per SD increase in TyG was associated with a 40% higher odds of RA (OR = 1.40; 95% CI: 1.15–1.70; *p* = 0.001). Similar positive associations were observed for all obesity-related TyG indices. Higher LE8 scores were inversely associated with RA. Each 10-point increase in LE8 was associated with a 26% lower odds of RA (95% CI: 0.67–0.94; *p* < 0.001). RCS analyses indicated linear associations of TyG-related indices and LE8 with the risk of RA (all *P*-nonlinear > 0.05). Mediation analyses suggested that TyG-related indices statistically accounted for a substantial proportion of the inverse association between LE8 and RA, with mediated proportions ranging from 32.8 to 67.3%, consistent with a pattern of statistical full mediation.

**Conclusion:**

Higher TyG-related indices were associated with an increased risk of RA, whereas better CVH was associated with a lower risk. These findings underscore the importance of CVH in early retinal microvascular injury and support the potential role of TyG-related indices as simple risk markers.

## Introduction

Retinal arteriosclerosis (RA) is a common microvascular abnormality characterized by narrowing and sclerosis of retinal arterioles (1). As a visible manifestation of systemic vascular injury, RA reflects the cumulative impact of cardiovascular risk factors and may serve as an early marker of cardiovascular and metabolic disease burden (2, 3). However, the metabolic pathways through which cardiovascular risk factors contribute to microvascular damage remain poorly understood.

Insulin resistance (IR) is a core feature of the cardiovascular-metabolic syndrome and an established early driver of cardiovascular disease (4, 5). Beyond its direct pathogenic role, IR is increasingly recognized as a mediator linking traditional cardiovascular risk factors to vascular dysfunction. Despite this importance, IR is underutilized in risk assessment due to the limitations of conventional diagnostic approaches (6, 7). The triglyceride-glucose (TyG) index, calculated from fasting triglycerides and glucose, has emerged as a simple and reliable surrogate marker of IR (8, 9). IR is closely intertwined with adiposity, particularly visceral fat accumulation, which amplifies metabolic dysregulation through inflammatory activation, free fatty acid flux, and adipokine imbalance (10). Moreover, TyG-related indices that integrate anthropometric measures such as BMI, waist circumference, and waist-to-height ratio provide enhanced capture of the combined effects of IR, adiposity, and dyslipidemia, and show superior predictive performance for macrovascular disease compared with the TyG index alone (11–15). However, whether these indices are associated with retinal arteriosclerosis (RA), remains unclear.

In 2022, the American Heart Association introduced the Life’s Essential 8 (LE8) framework to comprehensively quantify cardiovascular health (CVH) (16, 17). Higher LE8 scores have consistently been associated with reduced risks of cardiovascular disease and mortality (18, 19). Yet, it is unknown whether the protective effects of optimal CVH extend to microvascular injury such as RA.

Although healthcare workers are generally assumed to have better health literacy, they are frequently exposed to cardiometabolic risks due to shift work, psychosocial stress, physical inactivity, and insufficient sleep (20). Studies from Spain, Malaysia, and Mexico, indicate that this population experiences substantial cardiometabolic burden, with high rates of IR and metabolic syndrome (21–24). In our prior study, nearly 90% of male and 60% of female healthcare staff had already reached stage 1 or higher of the cardiovascular-kidney-metabolic syndrome (25). To address this, we established the Healthcare Employee Atherosclerosis Risk Tracking (HEART) cohort for early vascular risk evaluation.

Based on this cohort, the present cross-sectional study aimed to examine the associations of LE8, TyG-related indices with RA in a healthcare staff population, and to explore the potential statistical mediation of TyG-related indices in the relationship between CVH and RA.

## Methods

### Study design and population

**Figure 1:**
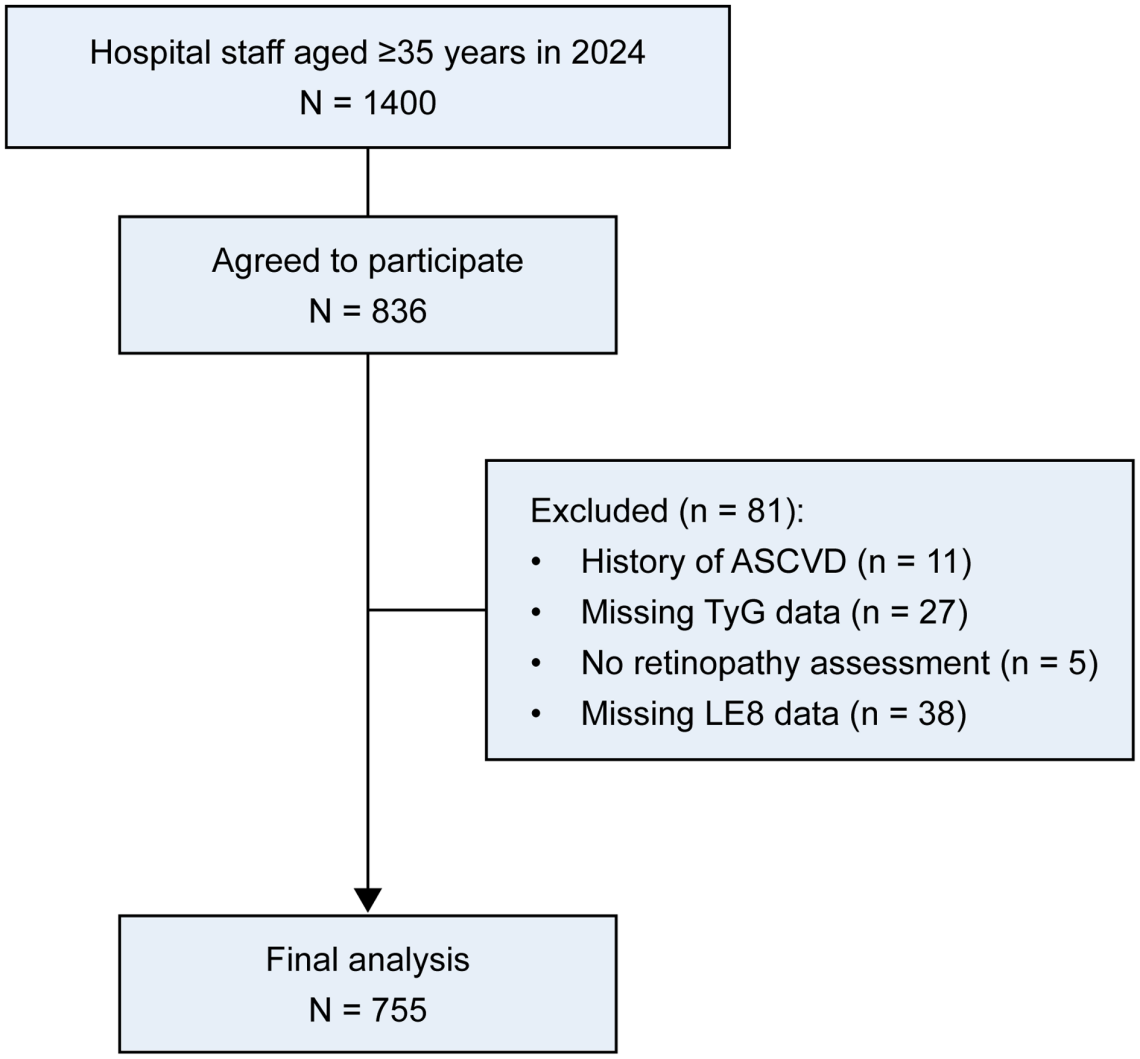
Flowchart of participant selection. ASCVD, Atherosclerotic cardiovascular disease; TyG, Triglyceride-glucose index.

This study was based on a cohort of healthcare staff aged 35 years or older who underwent routine pan-vascular risk assessment at the Vascular Disease Management Center of the Affiliated Taizhou People’s Hospital of Nanjing Medical University. According to hospital records, there were 1,400 staff members aged ≥ 35 years in 2024, of whom 836 agreed to participate in the cohort study. We conducted a cross-sectional study among healthcare staff aged 35 years or older between September and October 2024 to examine the associations of RA with TyG-related indices, and CVH as assessed by the LE8 score. After excluding 11 individuals with a history of atherosclerotic cardiovascular disease (ASCVD), 27 with missing TyG-related data, 5 with missing RA assessment, and 38 with missing key covariates, 755 participants were included in the final analysis (Figure 1). The study was approved by the Ethics Research Committee of Taizhou People’s Hospital. This study was conducted in accordance with the ethical principles outlined in the Declaration of Helsinki. All participants provided informed consent before the study began. The study was registered at: http://clinicaltrials.gov/, NCT number: NCT06545799. The registration covered the overall cohort design, study population, exposure assessment, and primary outcomes related to cardiometabolic and vascular health. The present analysis represents an observational, hypothesis-driven analysis nested within the registered cohort. Mediation analyses were conducted as exploratory *post hoc* analyses.

## Data collection

### Demographic data

General information, including medical history, medication use (antihypertensive, lipid-lowering, glucose-lowering, or antiplatelet drugs), and smoking and alcohol consumption habits, was collected using a standardized questionnaire. Smoking status was classified as current, former, or never smoking, with current smoking defined as smoking at least one cigarette per day for a minimum of 6 months. Drinking status was recorded as yes or no, with current drinking defined as consuming alcohol at least once per week for a duration of 6 months or more. Anthropometric measurements included height, weight, and waist circumference (WC), measured at the midpoint between the lower rib and iliac crest. Blood pressure was measured three times at two-minute intervals in the sitting position after at least 10 min of rest, and the average was used. Hypertension was defined as a self-reported history, use of antihypertensive medications, or systolic blood pressure (SBP) ≥ 140 mmHg and/or diastolic blood pressure (DBP) ≥ 90 mmHg. Dyslipidemia was defined as a self-reported history, lipid-lowering treatment, or abnormal lipid parameters (low-density lipoprotein cholesterol [LDL-C] ≥ 4.14 mmol/L, triglycerides [TG] ≥ 2.26 mmol/L, total cholesterol [TC] ≥ 6.22 mmol/L, or high-density lipoprotein cholesterol [HDL-C] < 1.04 mmol/L). Diabetes mellitus was defined as a self-reported history of diabetes, current use of glucose-lowering medication, or newly diagnosed diabetes according to standard diagnostic criteria (26). In individuals with classic symptoms, diabetes was diagnosed if any one of the following criteria was met: fasting plasma glucose (FPG) ≥ 7.0 mmol/L, 2-h OGTT glucose ≥ 11.1 mmol/L, random plasma glucose ≥ 11.1 mmol/L, or glycated hemoglobin (HbA1c) ≥ 6.5%. In the absence of classic symptoms, diagnosis required repeat testing on a different day or two different tests at the same visit (excluding random plasma glucose), with both results meeting or exceeding the diagnostic cutoffs. Atherosclerotic cardiovascular disease (ASCVD) was defined as a self-reported history of coronary heart disease, ischemic stroke, or peripheral artery disease. Self-reported medical history and medication use were further verified through the hospital electronic medical record system.

### Laboratory tests

Venous blood samples were drawn from participants after an overnight fast. Various biological markers, including TC, TG, LDL-C, HDL-C, FBG, and HbA1c, were measured.

### Calculation of TyG-related indices

We computed five TyG-related indices—TyG, TyG-BMI, TyG-WC, TyG-BRI, and TyG-WHtR. For the calculation of TyG, FPG and TG concentrations, originally measured in mmol/L, were converted to mg/dL using standard conversion factors (FPG: 1 mmol/L = 18 mg/dL; TG: 1 mmol/L = 88.57 mg/dL) prior to computation. The calculations for these indices were performed as follows (27):


TyGindex=ln[TG(mg/dl)×glucose(mg/dl)/2]

$$\mathrm{TyG}-\mathrm{BMI}=\mathrm{TyG}\;\text{index}\times [\text{weight}\;(\mathrm{kg})/{\text{height}}^{2}\;(m^{2})]$$

TyG−WC=TyGindex×WC(cm)

$$\begin{array}{l} \mathrm{TyG}-\mathrm{BRI}=\mathrm{TyG}\;\text{index}\\ \times [364.2-365.5\times \sqrt{1-[\mathrm{WC}(m)}/2\pi {]}^{2}/{\left[0.5\times \text{height}(m)\right]}^{2}] \end{array}$$

TyG−WHtR=TyGindex×[WC(cm)/height(cm)]

### Evaluation of RA

Non-mydriatic, macula-centered fundus photographs were obtained by experienced technicians using a non-mydriatic retinal camera (AFC-330, NIDEK Co., Ltd., Japan). Retinal microvascular abnormalities were assessed using the Keith-Wagener-Barker classification system, which categorizes retinal changes into five grades: grade 0 (normal, no abnormalities); grade 1 (mild to moderate generalized narrowing or sclerosis of the retinal arterioles); grade 2 (moderate to marked arteriolar sclerosis, focal narrowing, or signs of arteriosclerotic retinopathy or retinal vein thrombosis); grade 3 (angiospastic retinopathy with retinal edema, cotton-wool spots, and hemorrhages, in addition to marked arteriolar sclerosis); and grade 4 (optic disk edema accompanied by grade 3 features). In this study, RA was defined as a Keith-Wagener-Barker grade ≥ 1.

Retinal photographs were graded for RA by trained readers according to a standardized protocol, with graders blinded to participants’ clinical data. Each image was independently assessed by two experienced readers, and discrepancies were resolved by adjudication from a senior ophthalmologist. For analysis, the eye with the more severe lesion was used as the outcome.

### Evaluation of LE8

CVH was assessed using the LE8 framework proposed by the American Heart Association in 2022. LE8 comprises four health behaviors (diet, physical activity, smoking status, and sleep duration) and four health factors (BMI, blood pressure, blood lipids, and blood glucose) (17). Each component was scored on a scale from 0 to 100 according to AHA-recommended criteria, with higher scores indicating more favorable CVH. Behavioral components were derived from standardized self-administered questionnaires, while health factors were obtained from physical examinations and laboratory measurements. The overall LE8 score was calculated as the unweighted average of the eight component scores (range: 0–100). Participants were categorized as having optimal CVH (LE8 score ≥ 80) or suboptimal CVH (LE8 score < 80), consistent with prior studies. Detailed definitions, measurement procedures, and scoring algorithms for each LE8 component are provided in Supplementary Table 2.

### Statistical analysis

All statistical analyses were performed with R software (version 4.3.1). Continuous variables were expressed as mean ± standard deviation (SD) and analyzed using t-tests. Categorical data were presented as frequency and percentage (%) and analyzed using chi-square tests. Multicollinearity among covariates was assessed using the variance inflation factor (VIF). Binary logistic regression analysis was performed to assess the associations of the TyG index and its related obesity indices with RA, using both per SD increase and quartile categories. Three models were constructed: Model 1 was unadjusted; Model 2 was adjusted for age, sex, smoking status, drinking status, physical activity, sleep duration and diet scores; and Model 3 was further adjusted for medication use, including antihypertensive, lipid-lowering, glucose-lowering, and antiplatelet medications. Similarly, the association between LE8 score and RA was evaluated per 10-point increase and by CVH categories, classified as optimal (score ≥ 80) and suboptimal (score < 80). Two models were constructed: Model 1 was unadjusted; Model 2 was adjusted for age, sex, and drinking status. Restricted cubic spline (RCS) regression models were further used to explore potential non-linear dose–response relationships of LE8, the TyG-related indices with RA. RCS models with four knots (5th, 35th, 65th, and 95th percentiles) were fitted, using the median value as the reference. RCS analyses for LE8 and TyG-related indices were adjusted using the fully adjusted model. Subgroup analyses were conducted to assess potential effect modification according to sex and hypertension status. Sensitivity analyses were conducted by (1) additionally adjusting for SBP, DBP, HbA1c, and non-HDL-C to evaluate the robustness of the observed associations, and (2) estimating prevalence ratios (PRs) using modified Poisson regression models with a log link and robust variance estimator as an alternative effect measure. An exploratory mediation analysis was conducted to examine whether the TyG index and its obesity-related derivatives mediated the association between LE8 and RA. LE8 (per 10-point increase) was specified as the exposure (X), each TyG-related index (per SD increase) as the mediator (M), and RA as the outcome (Y). The mediator was modeled as a continuous variable using linear regression, and the binary outcome was modeled using logistic regression. Age and sex were included as covariates in both the mediator and outcome models. Indirect (ACME), direct (ADE), and total effects were estimated using the “mediation” package in R. Statistical uncertainty was assessed using nonparametric bootstrap resampling with 5,000 simulations. Statistical significance was set at two-tailed *p* < 0.05.

## Results

### Characteristics of the participants

A total of 755 participants were included, of whom 159 (21.06%) had RA. Compared with those without RA, these participants were older (45.48 vs. 43.81 years, *p* = 0.015) and more likely to be male (33.3% vs. 21.0%, *p =* 0.002). The RA group also had higher proportions of smokers (*p* = 0.004), alcohol drinkers (*p* < 0.001), and individuals with hypertension (19.5% vs. 8.7%, *p* < 0.001) and dyslipidemia (50.9% vs. 37.4%, *p* = 0.003). No significant differences were observed in education level, profession, or years of work experience between groups (*p* > 0.05). Participants with RA had significantly higher SBP, DBP, BMI, waist circumference, BRI and WHtR (all *p* < 0.001). In terms of laboratory findings, the RA group had higher FPG, HbA1c, LDL-C, and TG, while HDL-C was lower (all *p* < 0.001). TyG and related indices, including TyG-WC, TyG-BRI, TyG-WHtR, and TyG-BMI, were all significantly higher in the RA group (all *p* < 0.001). RA participants were more likely to have suboptimal CVH (78.6% vs. 65.9%), while those without RA had higher rates of optimal CVH (34.1% vs. 21.4%; *p* = 0.004). Moreover, the RA group had significantly lower LE8 scores (70.4 ± 11.8 vs. 74.3 ± 10.8, *p* < 0.001; Table 1).

**Table 1:** Baseline characteristics of participants according to the presence of RA.

Variable	Overall (*n* = 755)	RA		*p* value
		Present (*n* = 159)	Absent (*n* = 596)	
Age, years	44.26 ± 7.48	45.48 ± 8.26	43.82 ± 7.13	0.015
Gender				0.002
Female	577 (76.42)	106 (66.7)	471 (79.0)	
Male	178 (23.58)	53 (33.3)	125 (21.0)	
Education, %				0.521
Associate or bachelor	579 (76.7)	125 (78.6)	454 (75.9)	
Postgraduate or above	176 (23.3)	34 (21.4)	142 (23.7)	
Profession, %				0.079
Physicians	216 (28.6)	51 (32.1)	165 (27.7)	
Health technicians	94 (12.5)	17 (10.7)	77 (12.9)	
Nurses	331 (43.8)	59 (37.1)	272 (45.6)	
Non-clinical staff	114 (15.1)	32 (20.1)	82 (13.8)	
Years of work experience, %				0.060
<10	39 (5.2)	11 (6.9)	28 (4.7)	
10–20	316 (41.9)	54 (34.0)	262 (44.0)	
>20	400 (53.0)	94 (59.1)	306 (51.3)	
Smoking status, *n* (%)				0.004
No	691 (91.5)	134 (84.3)	557 (93.5)	
Former	19 (2.5)	8 (5.0)	17 (2.8)	
Current	45 (6.0)	17 (10.7)	22 (3.7)	
Drinking status, *n* (%)				<0.001
No	677 (89.7)	133 (83.7)	544 (91.3)	
Yes	78 (10.3)	26 (16.3)	52 (8.7)	
Hypertension, *n* (%)				<0.001
No	672 (89.0)	128 (80.5)	544 (91.3)	
Yes	83 (11.0)	31 (19.5)	52 (8.7)	
Dyslipidemia, *n* (%)				0.003
No	451 (59.7)	78 (49.1)	373 (62.6)	
Yes	304 (40.3)	81 (50.9)	223 (37.4)	
Medication use, *n* (%)				0.286
No	705 (93.4)	145 (91.2)	560 (94.0)	
Yes	50 (6.6)	14 (8.8)	36 (6.0)	
LE8	73.5 ± 11.09	70.4 ± 11.8	74.3 ± 10.8	<0.001
CVH Group				0.004
Suboptimal CVH (LE 8 < 80)	518 (68.6)	125 (78.6)	393 (65.9)	
Optimal CVH (LE 8 ≥ 80)	237 (31.4)	34 (21.4)	203 (34.1)	
SBP, mmHg	117.10 ± 14.23	122.06 ± 14.03	115.78 ± 9.64	<0.001
DBP, mmHg	70.57 ± 10.01	73.55 ± 11.17	69.78 ± 9.64	<0.001
BMI, kg/m^2^	23.97 ± 3.19	25.04 ± 3.23	23.68 ± 3.12	<0.001
Waist circumference, cm	81.86 ± 10.77	85.84 ± 10.15	80.79 ± 10.78	<0.001
BRI	3.48 ± 1.05	3.85 ± 1.03	3.39 ± 1.03	<0.001
WHTr	0.50 ± 0.06	0.52 ± 0.05	0.50 ± 0.06	<0.001
FPG, mmol/L	5.19 ± 0.86	5.38 ± 1.09	5.14 ± 0.78	0.009
HbA1c, %	5.64 ± 0.50	5.74 ± 0.66	5.61 ± 0.45	0.014
LDL-C, mmol/L	3.11 ± 0.69	3.23 ± 0.74	3.08 ± 0.68	0.028
TG, mmol/L	1.37 ± 0.94	1.62 ± 1.07	1.30 ± 0.89	<0.001
TC, mmol/L	4.95 ± 0.92	5.06 ± 0.97	4.92 ± 0.90	0.104
HDL-C, mmol/L	1.43 ± 0.29	1.37 ± 0.27	1.45 ± 0.29	<0.001
TyG	8.47 ± 0.59	8.67 ± 0.60	8.42 ± 0.57	<0.001
TyG-WC	696.38 ± 123.17	747.09 ± 124.46	682.85 ± 119.34	<0.001
TyG-BRI	29.79 ± 10.18	33.64 ± 10.49	28.77 ± 9.86	<0.001
TyG-WHTr	4.28 ± 0.69	4.55 ± 0.66	4.20 ± 0.68	<0.001
TyG-BMI	203.88 ± 36.11	217.84 ± 37.71	200.16 ± 34.77	<0.001

Data are mean ± SD, or *n* (%) SBP, systolic blood pressure; DBP, diastolic blood pressure; HbA1c, glycated hemoglobin; BMI, body mass index; BRI, body roundness index; WHtR, waist-to-height ratio; FPG, fasting plasma glucose; LDL-C, low-density lipoprotein cholesterol; TG, triglycerides; TC, total cholesterol; HDL-C, high-density lipoprotein cholesterol; TyG, triglyceride-glucose index.

### Association of TyG, TyG-BMI, TyG-WC, TyG-WHtR and TyG-BRI with RA

**Figure 2:**
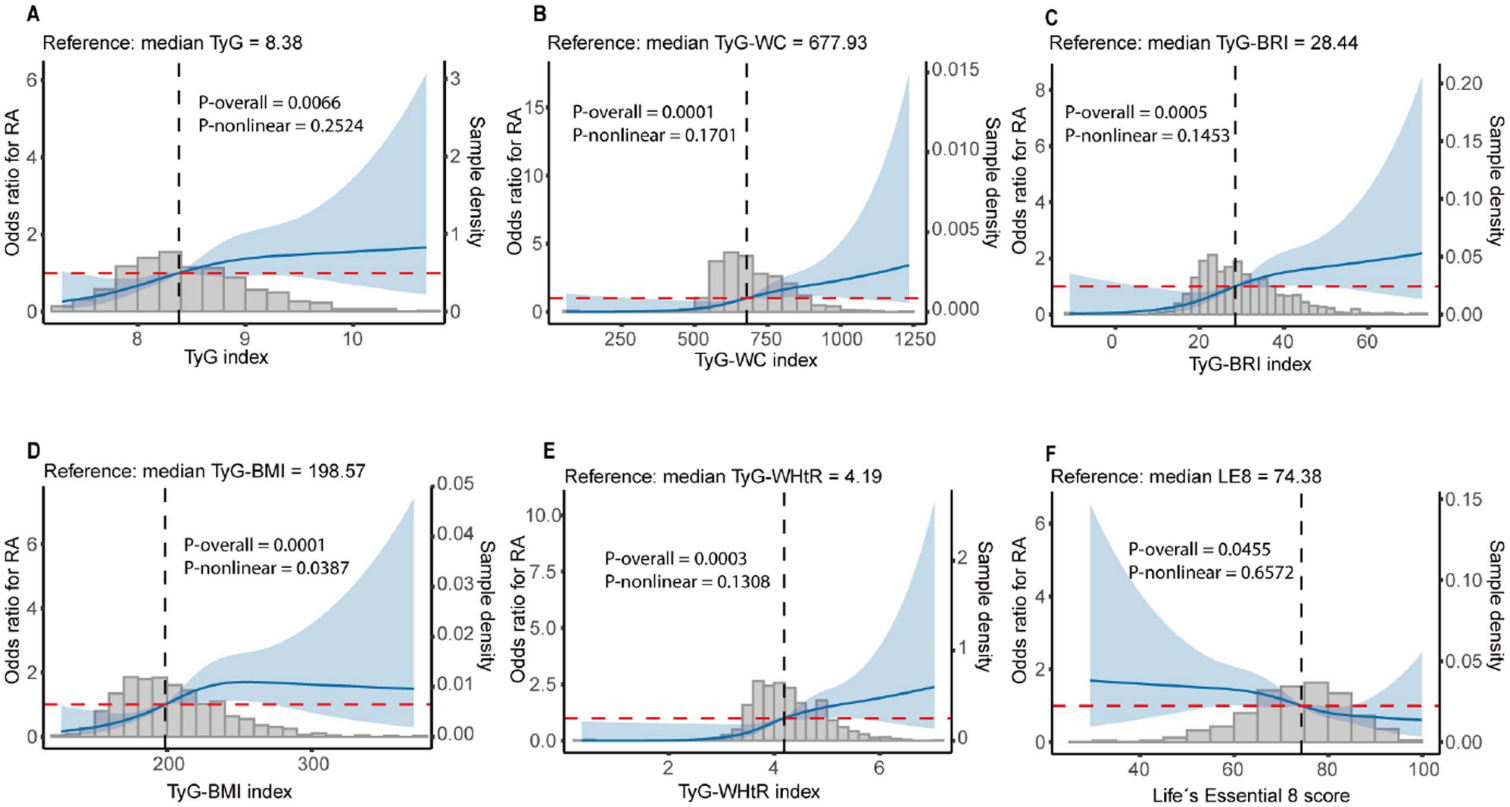
RCS analyses of TyG-related indices, LE 8 score, and risk of RA. **(A)** Association between the TyG index and RA. **(B)** Association between the TyG-WC index and RA. **(C)** Association between the TyG-BRI index and RA. **(D)** Association between the TyG-BMI index and RA. **(E)** Association between the TyG-WHtR index and RA. **(F)** Association between the Life’s Essential 8 (LE8) score and RA. The solid blue lines represent adjusted odds ratios (ORs), and the shaded areas represent 95% confidence intervals (CIs). The dashed vertical lines indicate the reference values (median). Histograms represent the distribution of each index in the study population.

As shown in Table 2, higher TyG-related indices were significantly associated with an increased risk of RA. In Model 3, per SD increase in TyG was associated with a 40% higher odds of RA (OR = 1.40; 95% CI: 1.15–1.70; *p* = 0.001). Similar positive associations were observed for all obesity-related TyG indices: TyG-WC (OR = 1.70; 95% CI: 1.36–2.13; *p* < 0.001), TyG-BRI (OR = 1.51; 95% CI: 1.25–1.83; *p* < 0.001), TyG-WHtR (OR = 1.60; 95% CI: 1.30–1.97; *p* < 0.001), and TyG-BMI (OR = 1.54; 95% CI: 1.27–1.87; *p* < 0.001). When TyG-related indices were categorized into quartiles, participants in higher quartiles (Q2–Q4) showed progressively elevated odds of RA compared with Q1, with all *P* for trend < 0.05. RCS analyses indicated linear associations of TyG-related indices with the risk of RA, with no evidence of significant nonlinearity (all *P*-nonlinear > 0.05, Figure 2).

**Table 2:** Associations of TyG-related indices with RA.

Variable	Model 1		Model 2		Model 3	
	OR (95% CI)	*p* value	OR (95% CI)	*p* value	OR (95% CI)	*p* value
TyG						
Per SD increase	1.50 (1.27–1.78)	<0.001	1.38 (1.14–1.67)	0.001	1.40 (1.15–1.70)	0.001
Q1	Ref		Ref		Ref	
Q2	1.62 (0.92–2.84)	0.093	1.53 (0.87–2.71)	0.139	1.57 (0.89–2.77)	0.122
Q3	1.86 (1.07–3.23)	0.028	1.60 (0.90–2.82)	0.107	1.64 (0.93–2.91)	0.088
Q4	3.12 (1.84–5.29)	<0.001	2.47 (1.40–4.36)	0.002	2.54 (1.43–4.53)	0.002
*p* for trend		<0.001		0.003		0.002
TyG-WC						
Per SD increase	1.69 (1.41–2.03)	<0.001	1.67 (1.34–2.08)	<0.001	1.70 (1.36–2.13)	<0.001
Q1	Ref		Ref		Ref	
Q2	1.38 (0.76–2.49)	0.293	1.33 (0.73–2.41)	0.353	1.33 (0.73–2.42)	0.349
Q3	2.68 (1.54–4.64)	<0.001	2.47 (1.40–4.37)	0.002	2.53 (1.43–4.48)	0.001
Q4	3.45 (2.01–5.92)	<0.001	2.93 (1.59–5.38)	0.001	3.02 (1.63–5.57)	<0.001
*p* for trend		<0.001		<0.001		<0.001
TyG-BRI						
Per SD increase	1.58 (1.33–1.88)	<0.001	1.48 (1.23–1.79)	<0.001	1.51 (1.25–1.83)	<0.001
Q1	Ref		Ref		Ref	
Q2	1.76 (0.95–3.23)	0.071	1.69 (0.92–3.13)	0.093	1.70 (0.92–3.14)	0.091
Q3	3.24 (1.83–5.76)	<0.001	2.87 (1.59–5.16)	<0.001	2.92 (1.62–5.26)	<0.001
Q4	4.06 (2.31–7.15)	<0.001	3.39 (1.87–6.17)	<0.001	3.51 (1.93–6.40)	<0.001
*p* for trend		<0.001		<0.001		<0.001
TyG-WHtR						
Per SD increase	1.67 (1.39–2.01)	<0.001	1.57 (1.28–1.92)	<0.001	1.60 (1.30–1.97)	<0.001
Q1	Ref		Ref		Ref	
Q2	1.49 (0.83–2.68)	0.184	1.43 (0.79–2.58)	0.236	1.44 (0.80–2.61)	0.224
Q3	2.39 (1.37–4.17)	0.002	2.08 (1.17–3.69)	0.013	2.15 (1.21–3.83)	0.009
Q4	3.62 (2.11–6.20)	<0.001	2.98 (1.66–5.33)	<0.001	3.08 (1.72–5.54)	<0.001
*p* for trend		<0.001		<0.001		<0.001
TyG-BMI						
Per SD increase	1.59 (1.34–1.89)	<0.001	1.52 (1.25–1.83)	<0.001	1.54 (1.27–1.87)	<0.001
Q1	Ref		Ref		Ref	
Q2	1.62 (0.87–3.01)	0.125	1.51 (0.81–2.82)	0.192	1.52 (0.81–2.83)	0.191
Q3	3.42 (1.93–6.06)	<0.001	3.06 (1.70–5.51)	<0.001	3.15 (1.75–5.68)	<0.001
Q4	4.06 (2.31–7.15)	<0.001	3.45 (1.89–6.30)	<0.001	3.52 (1.92–6.44)	<0.001
*p* for trend		<0.001		<0.001		<0.001

WC, waist circumference; BRI, body roundness index; WHtR, waist-to-height ratio; TyG, triglyceride-glucose index; OR, odds ratio; CI, confidence interval; RA, retinal arteriosclerosis. Model 1: Unadjusted. Model 2: Adjusted for age, sex, smoking status, drinking status, physical activity, sleep duration and diet scores. Model 3: Additionally adjusted for medication use, including antihypertensive, lipid-lowering, glucose-lowering, and antiplatelet medications.

### Association of CVH with RA

In multivariable analysis (Table 3), each 10-point increase in LE8 was associated with a 26% lower odds of RA in Model 1 (95% CI: 0.63–0.86; *p* < 0.001) and a 21% lower odds in Model 2 (95% CI: 0.67–0.94; *p* < 0.001). Participants with optimal CVH (LE8 ≥ 80) had significantly reduced risk compared with those with suboptimal CVH, with a 48% lower odds in Model 1 and 41% lower risk in Model 2. In the component analysis (Figure 2), optimal levels of lipid, HbA1c, blood pressure, BMI, and smoking were all significantly associated with a lower odds of RA. The ORs were 0.60 (95% CI: 0.42–0.86) for lipid, 0.57 (95% CI: 0.40–0.82) for HbA1c, 0.49 (95% CI: 0.34–0.70) for blood pressure, 0.47 (95% CI: 0.33–0.67) for BMI, and 0.38 (95% CI: 0.22–0.64) for smoking (all *p* < 0.01). RCS analyses indicated linear associations of LE8 with the risk of RA, with no evidence of significant nonlinearity (*P*-nonlinear = 0.701, Figure 2).

**Table 3:** Associations of CVH with RA.

Variable	Model 1		Model 2	
	OR (95% CI)	*p* value	OR (95% CI)	*p* value
LE 8 (Per 10 increase)	0.74 (0.63–0.86)	<0.001	0.79 (0.67–0.94)	<0.001
CVH group		0.002		0.015
Suboptimal CVH (LE 8 < 80)	Ref		Ref	
Optimal CVH (LE 8 ≥ 80)	0.52 (0.35–0.80)		0.59 (0.39–0.90)	

CVH, cardiovascular health; RA, retinal arteriosclerosis. Model 1: Unadjusted. Model 2: Adjusted for age, sex and drinking status.

### Subgroup and sensitivity analyses

To ensure the robustness of our findings, additional sensitivity and subgroup analyses were conducted. In the sensitivity analyses, the main results remained materially unchanged after further adjustment for additional cardiometabolic risk factors, including systolic blood pressure, diastolic blood pressure, HbA1c, and non–HDL cholesterol (Supplementary Table 3). Sensitivity analyses using modified Poisson regression to estimate prevalence ratios yielded results that were materially consistent with those obtained from logistic regression (Supplementary Table 4). Furthermore, we examined the associations between TyG-related indices and RA across predefined subgroups stratified by sex (male vs. female) and history of hypertension (yes vs. no) (Supplementary Table 4). No significant interactions were observed across these subgroups. Overall, the subgroup and sensitivity analyses yielded results consistent with those of the primary analyses, further supporting the robustness and stability of our conclusions.

### Mediation analysis

**Figure 3:**
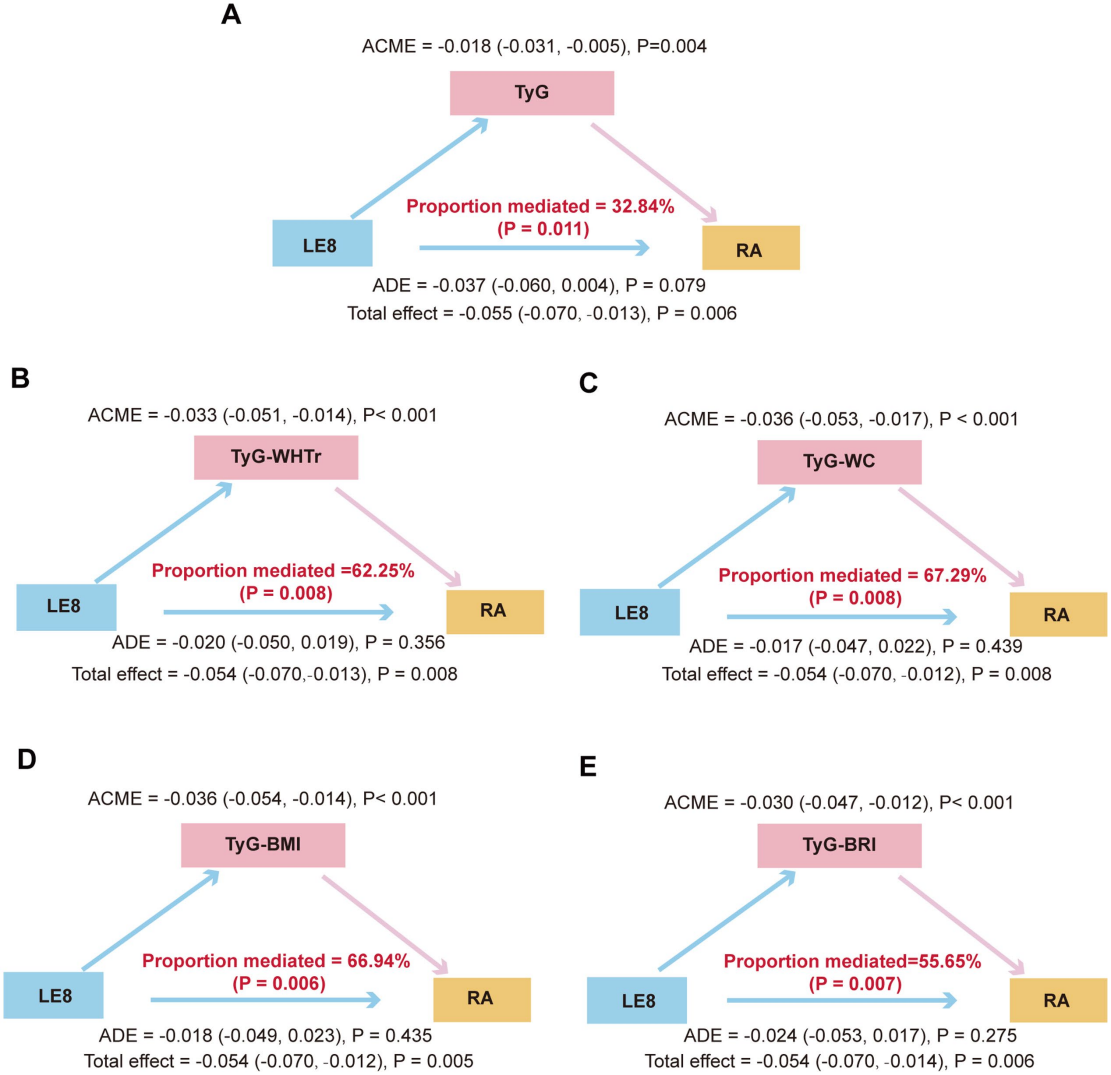
Mediation effects of TyG-related indices on the association between LE8 and RA. LE8, Life’s Essential 8; WC, waist circumference; BRI, body roundness index; WHtR, waist-to-height ratio; TyG, triglyceride-glucose index; OR, odds ratio; CI, confidence interval; RA, retinal arteriosclerosis. **(A)** Mediation analysis with the TyG index as the mediator. **(B)** Mediation analysis with the TyG-WHtR index as the mediator. **(C)** Mediation analysis with the TyG-WC index as the mediator. **(D)** Mediation analysis with the TyG-BMI index as the mediator. **(E)** Mediation analysis with the TyG-BRI index as the mediator. ACME represents the average causal mediation effect, ADE represents the average direct effect, and the proportion mediated indicates the percentage of the total effect explained by the mediator.

Figure 3 illustrates the mediation effects of TyG-related indices in the association between LE8 and the risk of RA. The TyG index significantly mediated the relationship between LE8 and RA, accounting for 32.84% of the total effect (*p* = 0.009). TyG-WHtR mediated 62.25% of the effect (*p* = 0.005), TyG-WC mediated 67.29% of the effect (*p* = 0.008), TyG-BMI mediated 66.94% of the effect (*p* = 0.006), TyG-BRI mediated 55.65% of the effect (*p* = 0.006). To minimize potential overadjustment or overlap arising from the inclusion of glycemic, lipid, and adiposity components within the LE8 score, we conducted an additional sensitivity mediation analysis by excluding the glucose, lipid, and BMI components from LE8 to derive a modified LE5 score. The results showed significant indirect effects of TyG-related indices in the association between LE5 and RA, whereas the proportion mediated was not statistically significant (Supplementary Figure 1).

## Discussion

To our knowledge, this is the first study to examine the associations of IR, represented by TyG-related indices, and overall CVH, measured by the LE8, with RA. Beyond documenting these associations, our findings provide novel evidence that the TyG index acts as a critical mediator linking cardiovascular risk factors to microvascular damage. In this cross-sectional study of healthcare staff, higher levels of TyG-related indices were significantly associated with an increased odds of RA, whereas higher LE8 scores were protective. RCS analyses further supported approximately linear dose–response relationships across the observed ranges of TyG-related indices and LE8 scores. Mediation analyses indicated that the association between LE8 and RA was fully mediated by TyG-related indices.

Prior studies have consistently demonstrated that the TyG index and its derivatives predict macrovascular outcomes, including ASCVD, stroke, and arterial stiffness (28–31). Our study extends this evidence to the microvascular domain, showing that both TyG and TyG-related obesity indices are significantly associated with RA. The enhanced predictive power of composite indices suggests that integrating obesity parameters with TyG may better capture the clustering of dyslipidemia, hyperglycemia, and central adiposity, which collectively drive early vascular injury. This aligns with findings from the Kailuan and CHARLS cohorts, where TyG-related indices showed superior predictive value for macrovascular outcomes compared with TyG alone (11, 32). A recent systematic review and meta-analysis demonstrated that both lipid levels and lipid variability are significantly associated with the risk of multiple microvascular complications in patients with diabetes, including nephropathy, neuropathy, and, in some studies, retinopathy (33). These findings suggest that dyslipidemia itself plays an important role in microvascular injury, thereby supporting our observation that higher TyG-related indices are associated with an increased risk of retinal arteriolar sclerosis. Nevertheless, given the cross-sectional design, reverse causality cannot be excluded, with microvascular damage potentially contributing to metabolic dysregulation and insulin resistance.

The mechanistic plausibility of our findings is supported by the established role of IR in promoting vascular injury (5, 34). The TyG index integrates fasting triglycerides and glucose, both of which contribute to endothelial dysfunction, oxidative stress, inflammation, and arterial stiffness (35, 36). These mechanisms can accelerate structural remodeling in both large and small arteries. In the microvascular context, such as the retina, IR may impair autoregulation, promote basement membrane thickening, and increase vascular permeability, ultimately leading to tissue ischemia and damage (37). When obesity-related measures are incorporated, the indices also capture the influence of visceral adiposity, which aggravates IR and promotes secretion of inflammatory cytokines such as TNF-*α* and IL-6, further compromising microvascular integrity (38).

The LE8 framework provides a comprehensive assessment of CVH and has been consistently validated as a robust predictor of adverse cardiovascular outcomes (19, 39, 40). In the present study, higher LE8 scores were significantly associated with a lower risk of RA, underscoring the importance of overall CVH for retinal microvascular integrity. Component-level analyses further revealed that lipid status, HbA1c, blood pressure, BMI, and smoking status were each independently associated with RA, representing some of the most important and modifiable contributors to the global burden of cardiovascular disease (41).

From a pathophysiological perspective, blood pressure-related microvascular remodeling and smoking-induced endothelial injury are well-established determinants of structural changes in retinal arteriolar sclerosis (42). A recent optical coherence tomography angiography study has additionally demonstrated that smoking is associated with impaired retinal microvascular reactivity and reduced perfused vessel density, supporting a direct role of tobacco exposure in early microvascular injury (43). Collectively, these observations support the biological plausibility of the inverse association between LE8 score and RA observed in our study and highlight the importance of integrated cardiometabolic risk control for preserving microvascular health. In this study, retinal arteriolar sclerosis was assessed using the Keith–Wagener–Barker classification, a grading system that has been widely applied in large-scale epidemiological studies to characterize structural changes of retinal arterioles (44). We acknowledge that early-stage changes (grade 1), characterized mainly by mild arteriolar narrowing, may partly reflect vascular aging. Nevertheless, such changes are often interpreted as reflecting cumulative microvascular remodeling associated with long-term exposure to cardiometabolic risk factors in population-based research (45).

Mediation analysis indicated that TyG-related indices contributed to the inverse association between LE8 and RA. When LE5 was used as the exposure, indirect effects remained statistically significant, although the mediated proportion did not reach significance, possibly due to limited statistical power. However, as both LE8 and TyG-related indices were assessed at baseline, the lack of prospective longitudinal data precludes the establishment of a clear temporal sequence, thereby limiting causal inference regarding the directionality of these pathways. Consequently, the observed mediating relationships should be interpreted with caution and warrant confirmation in future prospective studies incorporating repeated measurements of exposures, potential mediators, and microvascular outcomes to determine whether they reflect true causal mechanisms.

Our findings have important implications. TyG-related indices were consistently associated with RA, supporting their potential utility as simple and cost-effective markers for identifying individuals at higher risk of microvascular injury. In addition, higher LE8 scores were inversely associated with RA, highlighting the importance of overall CVH in early microvascular prevention. Mediation analyses further suggested that the protective association of LE8 with RA could be partly statistically explained by TyG-related indices. Several limitations should be acknowledged. First, this cross-sectional study was conducted among healthcare staff, which may limit generalizability; moreover, information on eligible but non-participating staff was unavailable, precluding comparison between included and non-included individuals and raising the possibility of selection bias. Second, although LE8 provides a comprehensive assessment of cardiovascular health, there is inevitable construct overlap between LE8 components (e.g., glucose, lipids, BMI) and TyG-related indices, which may inflate associations and constrain mechanistic interpretation, particularly in mediation analyses. Third, despite adjustment for multiple covariates, residual confounding cannot be excluded, especially from occupational factors such as shift work and job-related stress, which were not fully accounted for. Fourth, several variables were self-reported, raising the possibility of recall or misclassification bias. Fifth, the use of the Keith-Wagener-Barker classification, a semi-quantitative and observer-dependent system, may have limited sensitivity for identifying subtle or early retinal microvascular changes. Sixth, missing data were handled using complete-case analysis, which may have introduced bias if missingness was not random. Finally, all exposures and outcomes were assessed at baseline, precluding evaluation of temporal relationships. Additionally, the use of odds ratios derived from logistic regression in the context of a relatively common outcome may influence the magnitude of the estimated associations. Nevertheless, sensitivity analyses using prevalence ratios demonstrated consistent findings, supporting the robustness of our results.

## Conclusion

In this cross-sectional study of healthcare staff, higher levels of TyG-related indices were consistently associated with an increased risk of RA, whereas better overall CVH as assessed by LE8 was associated with a lower risk. Further analyses suggested that TyG-related indices played an important mediating role in the association between LE8 and RA. Collectively, these findings underscore the importance of comprehensive cardiometabolic risk control in the prevention of retinal microvascular injury and support the potential utility of TyG-related indices as simple and accessible markers for identifying individuals at high risk. Prospective studies with repeated measurements are warranted to clarify the temporal relationships and underlying causal mechanisms

## Data Availability

The raw data supporting the conclusions of this article will be made available by the authors, without undue reservation.
