# Supplementary material for: Associations of triglyceride-glucose-related indices and cardiovascular health with retinal arteriosclerosis: a cross-sectional study
Source: Front Public Health. 2026 Mar 16;14:1766122. doi: 10.3389/fpubh.2026.1766122 (PMC13033492; doi:10.3389/fpubh.2026.1766122)
Supplement: Supplementary file 1 [file Table_1.DOCX]

**Additional File 1**

**Supplementary Table 1:** Variance inflation factors for covariates included in the multivariable models

**Supplementary Table 2:** Definitions, measurement methods, and scoring criteria of Life’s Essential 8 cardiovascular health metrics

**Supplementary Table 3:** Sensitivity analyses were conducted to evaluate the associations between TyG-related indices and RA after further adjustment for additional cardiometabolic risk factors, including SBP, DBP, HbA1c, and non-HDL cholesterol

**Supplementary Table 4:** Sensitivity analyses using prevalence ratios for the associations between TyG-related indices and retinal arteriosclerosis

**Supplementary Table 5** Subgroup analysis of the association between TyG-related indices and RA.

**Supplementary Figure 1:** Mediation effects of TyG-related indices on the association between LE5 and RA.

**Supplementary Table 1.** Variance inflation factors for covariates included in the multivariable models

| variables | VIF |
| --- | --- |
| Age | 1.095 |
| Gender | 1.336 |
| Smoking status | 1.069 |
| Drinking status | 1.257 |
| Diet scores | 1.025 |
| Sleep duration | 1.025 |
| Physical activity | 1.043 |
| Medication use | 1.15 |
| HbA1c | 1.319 |
| non-HDL-C | 1.052 |
| SBP | 1.691 |
| DBP | 1.705 |

VIFs, Variance inflation factors; SBP, systolic blood pressure; DBP, diastolic blood pressure; HDL, high-density lipoprotein cholesterol.

**Supplementary Table 2.** Definitions, measurement methods, and scoring criteria of Life’s Essential 8 cardiovascular health metrics

| Domain | CVH metric | Method of measurement | Quantification of CVH metric |
| --- | --- | --- | --- |
| Health behaviors | Diet | Self-reported daily intake of a DASH-style eating pattern | Diet scored based on population percentiles.Participants at or above the 95th percentile received 100 points; those in the 75th–94th percentile received 80 points; those in the 50th–74th percentile received 50 points; those in the 25th–49th percentile received 25 points; and those in the 1st–24th percentile received 0 points. |
|  | Physical Activity | Self-reported minutes of moderate or vigorous physical activity per week | Participants performing ≥150 minutes per week received 100 points; 120–149 minutes received 90 points; 90–119 minutes received 80 points; 60–89 minutes received 60 points; 30–59 minutes received 40 points; 1–29 minutes received 20 points; and no physical activity received 0 points. |
|  | Nicotine exposure | Self-reported use of cigarettes or inhaled nicotine-delivery system | Participants who never smoked received 100 points; former smokers who quit ≥5 years ago received 75 points; those who quit 1–<5 years ago received 50 points; those who quit <1 year ago or used inhaled nicotine delivery systems received 25 points; and current smokers received 0 points. A 20-point deduction was applied for living with an active indoor smoker (minimum score = 0). |
|  | Sleep health | Self-reported average hours of sleep per night. | Participants sleeping 7–9 hours per night received 100 points; 9–10 hours received 90 points; 6–7 hours received 70 points; 5–6 hours or more than 10 hours received 40 points; 4–5 hours received 20 points; and less than 4 hours received 0 points. |
| Health factors | BMI | Body weight (kilograms) divided by height squared (meters squared) | Participants with BMI <25.0 received 100 points; BMI 25.0–29.9 received 70 points; BMI 30.0–34.9 received 40 points; BMI 35.0–39.9 received 20 points; and BMI ≥40.0 received 0 points. |
|  | Blood Lipids | Plasma total and HDL cholesterol with calculation of non–HDL cholesterol | Participants with non–HDL cholesterol <130 mg/dL received 100 points; 130–159 mg/dL received 60 points; 160–189 mg/dL received 40 points; 190–219 mg/dL received 20 points; and ≥220 mg/dL received 0 points. Lipid-lowering medication use resulted in a 20-point deduction, with a minimum score of 0. |
|  | Blood Glucose | FBG and HbA1c | Participants without diabetes and with FPG <100 mg/dL or HbA1c <5.7% received 100 points; those without diabetes but with FPG 100–125 mg/dL or HbA1c 5.7–6.4% (prediabetes) received 60 points. Participants with diabetes received 40 points if HbA1c was <7.0%, 30 points for HbA1c 7.0–7.9%, 20 points for HbA1c 8.0–8.9%, 10 points for HbA1c 9.0–9.9%, and 0 points for HbA1c ≥10.0%. |
|  | Blood Pressure | SBP and DBP | Participants with BP <120/<80 mmHg received 100 points; those with SBP 120–129 mmHg and DBP <80 mmHg received 75 points; SBP 130–139 mmHg or DBP 80–89 mmHg received 50 points; SBP 140–159 mmHg or DBP 90–99 mmHg received 25 points; and SBP ≥160 mmHg or DBP ≥100 mmHg received 0 points. Use of antihypertensive medication resulted in a 20-point deduction, with the minimum score capped at 0. |

SBP, systolic blood pressure; DBP, diastolic blood pressure; BMI, body mass index; FPG, fasting plasma glucose; HbA1c, glycated hemoglobin; DASH, Dietary Approaches to Stop Hypertension; HDL, high-density lipoprotein cholesterol; CVH, cardiovascular health.

**Supplementary Table 3.** Sensitivity analyses were conducted to evaluate the associations between TyG-related indices and retinal arteriosclerosis after further adjustment for additional cardiometabolic risk factors, including SBP, DBP, HbA1c, and non-HDL cholesterol.

|  | OR (95% CI) | *P* value |
| --- | --- | --- |
| TyG |  |  |
| Per SD increase | 1.40 (1.15–1.70) | 0.001 |
| Q1 | Ref |  |
| Q2 | 1.57 (0.89–2.77) | 0.122 |
| Q3 | 1.64 (0.93–2.91) | 0.088 |
| Q4 | 2.54 (1.43–4.53) | 0.002 |
| *P* for trend |  | 0.002 |
| TyG-WC |  |  |
| Per SD increase | 1.70 (1.36–2.13) | <0.001 |
| Q1 | Ref |  |
| Q2 | 1.33 (0.73–2.42) | 0.349 |
| Q3 | 2.53 (1.43–4.48) | 0.001 |
| Q4 | 3.02 (1.63–5.57) | <0.001 |
| *P* for trend |  | <0.001 |
| TyG-BRI |  |  |
| Per SD increase | 1.51 (1.25–1.83) | <0.001 |
| Q1 | Ref |  |
| Q2 | 1.70 (0.92–3.14) | 0.091 |
| Q3 | 2.92 (1.62–5.26) | <0.001 |
| Q4 | 3.51 (1.93–6.40) | <0.001 |
| *P* for trend |  | <0.001 |
| TyG-WHtR |  |  |
| Per SD increase | 1.60 (1.30–1.97) | <0.001 |
| Q1 | Ref |  |
| Q2 | 1.44 (0.80–2.61) | 0.224 |
| Q3 | 2.15 (1.21–3.83) | 0.009 |
| Q4 | 3.08 (1.72–5.54) | <0.001 |
| *P* for trend |  | <0.001 |
| TyG-BMI |  |  |
| Per SD increase | 1.54 (1.27–1.87) | <0.001 |
| Q1 | Ref |  |
| Q2 | 1.52 (0.81–2.83) | 0.191 |
| Q3 | 3.15 (1.75–5.68) | <0.001 |
| Q4 | 3.52 (1.92–6.44) | <0.001 |
| *P* for trend |  | <0.001 |

BMI, body mass index; BRI, body roundness index; WHtR, waist-to-height ratio; TyG, triglyceride-glucose index.

**Supplementary Table 4.** Sensitivity analyses using prevalence ratios for the associations between TyG-related indices and RA

| Variable | Model 1 | |  | Model 2 | |  | Model 3 | |
| --- | --- | --- | --- | --- | --- | --- | --- | --- |
|  | PR (95% CI) | *P* value |  | PR (95% CI) | *P* value |  | PR (95% CI) | *P* value |
| TyG |  |  |  |  |  |  |  |  |
| Per SD increase | 1.35 (1.21–1.51) | <0.001 |  | 1.27 (1.11–1.44) | <0.001 |  | 1.28 (1.11–1.46) | <0.001 |
| Q1 | Ref |  |  | Ref |  |  | Ref |  |
| Q2 | 1.50 (0.93–2.41) | 0.095 |  | 1.44 (0.89–2.33) | 0.139 |  | 1.46 (0.90–2.36) | 0.123 |
| Q3 | 1.68 (1.05–2.66) | 0.029 |  | 1.49 (0.93–2.39) | 0.097 |  | 1.52 (0.95–2.44) | 0.081 |
| Q4 | 2.46 (1.60–3.78) | <0.001 |  | 2.04 (1.29–3.24) | 0.002 |  | 2.09 (1.31–3.32) | 0.002 |
| *P* for trend |  | <0.001 |  |  | 0.002 |  |  | 0.002 |
| TyG-WC |  |  |  |  |  |  |  |  |
| Per SD increase | 1.46 (1.31–1.62) | <0.001 |  | 1.45 (1.27–1.65) | <0.001 |  | 1.46 (1.28–1.67) | <0.001 |
| Q1 | Ref |  |  | Ref |  |  | Ref |  |
| Q2 | 1.32 (0.79–2.21) | 0.294 |  | 1.28 (0.77–2.15) | 0.344 |  | 1.29 (0.77–2.15) | 0.339 |
| Q3 | 2.24 (1.41–3.55) | 0.001 |  | 2.10 (1.31–3.37) | 0.002 |  | 2.14 (1.33–3.42) | 0.002 |
| Q4 | 2.68 (1.72–4.19) | <0.001 |  | 2.36 (1.46–3.82) | <0.001 |  | 2.41 (1.50–3.89) | <0.001 |
| *P* for trend |  | <0.001 |  |  | <0.001 |  |  | <0.001 |
| TyG-BRI |  |  |  |  |  |  |  |  |
| Per SD increase | 1.39 (1.26–1.54) | <0.001 |  | 1.33 (1.18–1.5) | <0.001 |  | 1.35 (1.19–1.52) | <0.001 |
| Q1 | Ref |  |  | Ref |  |  | Ref |  |
| Q2 | 1.63 (0.96–2.78) | 0.073 |  | 1.59 (0.93–2.69) | 0.088 |  | 1.59 (0.94–2.70) | 0.087 |
| Q3 | 2.65 (1.62–4.31) | <0.001 |  | 2.40 (1.46–3.95) | 0.001 |  | 2.44 (1.48–4.00) | <0.001 |
| Q4 | 3.11 (1.93–5.00) | <0.001 |  | 2.70 (1.65–4.42) | <0.001 |  | 2.76 (1.69–4.52) | <0.001 |
| *P* for trend |  | <0.001 |  |  | <0.001 |  |  | <0.001 |
| TyG-WHtR |  |  |  |  |  |  |  |  |
| Per SD increase | 1.45 (1.3–1.62) | <0.001 |  | 1.39 (1.22–1.58) | <0.001 |  | 1.41 (1.24–1.60) | <0.001 |
| Q1 | Ref |  |  | Ref |  |  | Ref |  |
| Q2 | 1.41 (0.85–2.34) | 0.186 |  | 1.37 (0.82–2.26) | 0.227 |  | 1.38 (0.83–2.28) | 0.215 |
| Q3 | 2.06 (1.29–3.28) | 0.003 |  | 1.85 (1.14–2.99) | 0.012 |  | 1.90 (1.18–3.07) | 0.009 |
| Q4 | 2.77 (1.78–4.32) | <0.001 |  | 2.39 (1.49–3.82) | <0.001 |  | 2.44 (1.53–3.91) | <0.001 |
| *P* for trend |  | <0.001 |  |  | <0.001 |  |  | <0.001 |
| TyG-BMI |  |  |  |  |  |  |  |  |
| Per SD increase | 1.40 (1.27–1.54) | <0.001 |  | 1.35 (1.20–1.52) | <0.001 |  | 1.36 (1.21–1.53) | <0.001 |
| Q1 | Ref |  |  | Ref |  |  | Ref |  |
| Q2 | 1.53 (0.89–2.62) | 0.126 |  | 1.45 (0.84–2.49) | 0.181 |  | 1.45 (0.84–2.50) | 0.179 |
| Q3 | 2.75 (1.69–4.47) | <0.001 |  | 2.52 (1.54–4.12) | <0.001 |  | 2.57 (1.57–4.19) | <0.001 |
| Q4 | 3.11 (1.93–5.00) | <0.001 |  | 2.74 (1.66–4.50) | <0.001 |  | 2.77 (1.68–4.57) | <0.001 |
| *P* for trend |  | <0.001 |  |  | <0.001 |  |  | <0.001 |

BMI, body mass index; BRI, body roundness index; WHtR, waist-to-height ratio; TyG, triglyceride-glucose index.

**Supplementary Table 5** Subgroup analysis of the association between TyG-related indices and RA.

|  | OR (95% CI) | P value | P interaction |
| --- | --- | --- | --- |
| TyG |  |  |  |
| Sex |  |  | 0.655 |
| Male | 1.36 (0.97–1.91) | 0.075 |  |
| Female | 1.38 (1.08–1.77) | 0.011 |  |
| Hypertension |  |  | 0.857 |
| No | 1.34 (1.07–1.67) | 0.011 |  |
| Yes | 2.53 (1.24–5.17) | 0.011 |  |
| TyG-WC |  |  |  |
| Sex |  |  | 0.849 |
| Male | 1.77 (1.19–2.65) | 0.005 |  |
| Female | 1.65 (1.25–2.18) | <0.001 |  |
| Hypertension |  |  | 0.746 |
| No | 1.62 (1.25–2.10) | <0.001 |  |
| Yes | 2.49 (1.27–4.89) | 0.008 |  |
| TyG-BRI |  |  |  |
| Sex |  |  | 0.897 |
| Male | 1.49 (1.05–2.10) | 0.025 |  |
| Female | 1.52 (1.20–1.93) | 0.001 |  |
| Hypertension |  |  | 0.636 |
| No | 1.46 (1.16–1.83) | 0.001 |  |
| Yes | 1.84 (1.08–3.11) | 0.024 |  |
| TyG-WHtR |  |  |  |
| Sex |  |  | 0.898 |
| Male | 1.60 (1.08–2.36) | 0.018 |  |
| Female | 1.59 (1.23–2.05) | <0.001 |  |
| Hypertension |  |  | 0.810 |
| No | 1.52 (1.20–1.94) | 0.001 |  |
| Yes | 2.26 (1.21–4.22) | 0.011 |  |
| TyG-BMI |  |  |  |
| Sex |  |  | 0.580 |
| Male | 1.43 (1.02–2.02) | 0.040 |  |
| Female | 1.59 (1.25–2.02) | <0.001 |  |
| Hypertension |  |  | 0.463 |
| No | 1.52 (1.21–1.90) | <0.001 |  |
| Yes | 1.96 (1.12–3.43) | 0.019 |  |

BMI, body mass index; BRI, body roundness index; WHtR, waist-to-height ratio; TyG, triglyceride-glucose index.

**
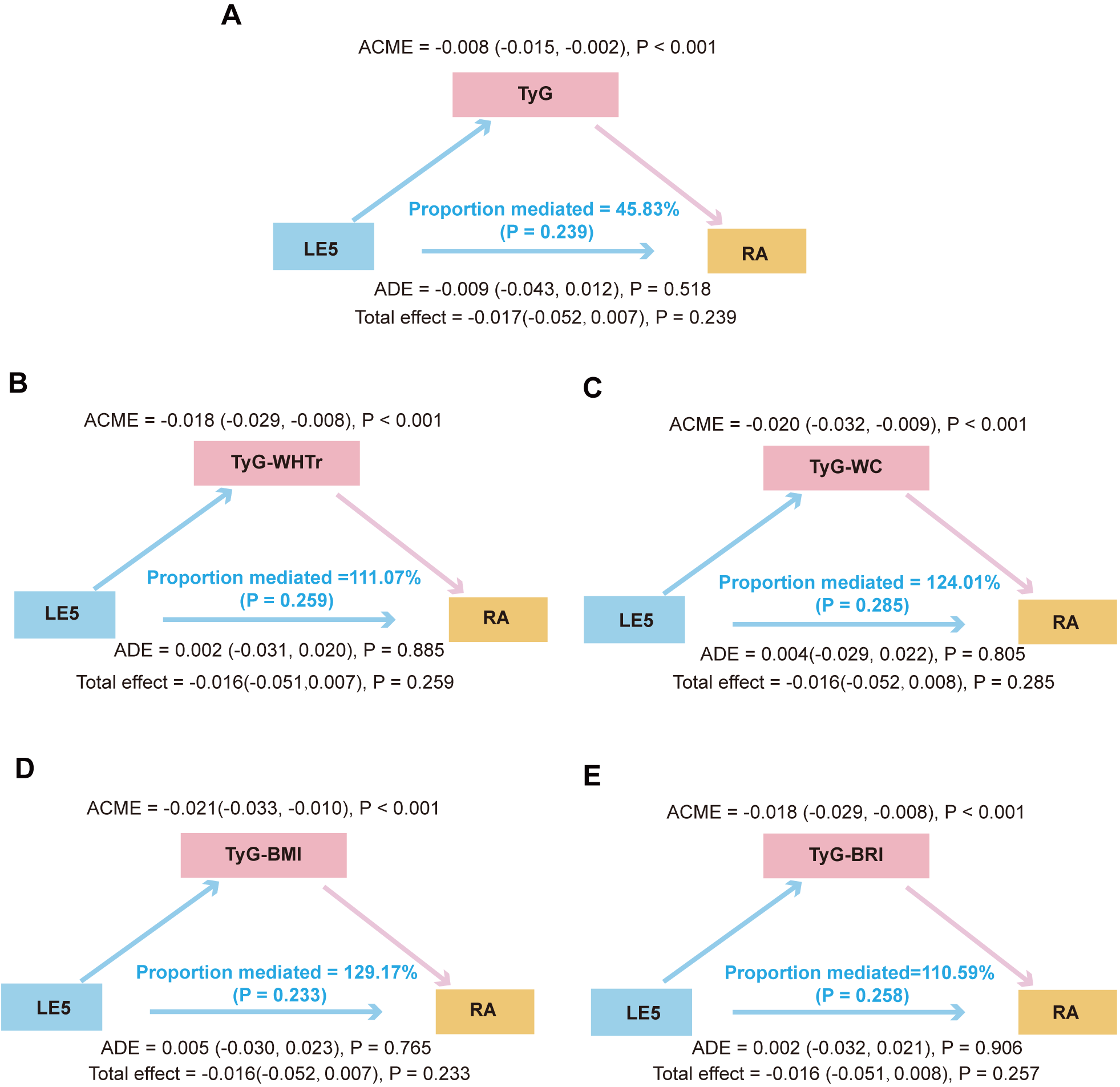
**

**Supplementary Figure 1.** Mediation effects of TyG-related indices on the association between LE5 and RA.
